# The rationale behind updates to ambient ozone guidelines and standards

**DOI:** 10.3389/fpubh.2023.1273826

**Published:** 2023-10-18

**Authors:** Kaibing Xue, Xin Zhang

**Affiliations:** ^1^College of Resources and Environment, University of Chinese Academy of Sciences, Beijing, China; ^2^Yanshan Critical Zong Nation Research Station, University of Chinese Academy of Sciences, Beijing, China

**Keywords:** ozone, air quality guidelines, WHO, National Ambient Air Quality Standards, EPA

## Abstract

Although air quality has gradually improved in recent years, as shown by the decrease in PM_2.5_ concentration, the problem of rising ambient ozone has become increasingly serious. To reduce hazards to human health and environmental welfare exposure to ozone, scientists and government regulators have developed ozone guidelines and standards. These answer the questions of which levels of exposure are hazardous to human health and the environment, and how can ambient ozone exposure be guaranteed, respectively. So what are the basis for the ozone guidelines and standards? This paper reviews in detail the process of revising ozone guidelines and standards by the World Health Organization (WHO) and the United States Environmental Protection Agency (EPA). The present study attempts to explore and analyze the scientific basis and empirical methods for updating guidelines and standards, in a view to guide the future revision process and provide directions for further scientific research. We found many epidemiological and toxicological studies and exposure-response relationships provided strong support for developing and revising the ozone guidelines. When setting standards, ozone exposure has been effectively considered, and the economic costs, health, and indirect economic benefits of standard compliance were reasonably estimated. Accordingly, epidemiological and toxicological studies and the establishment of exposure-response relationships, as well as exposure and risk assessment and benefit-cost estimates of standards compliance should be strengthened for the further update of guidelines and standards. In addition, with the increasing prominence of combined air pollution led by ozone and PM_2.5_, more joint exposure scientific research related to ozone guidelines and standards should be undertaken.

## 1. Introduction

Ozone is an important component of photochemical oxidants in the atmospheric environment and is listed as one of the important atmospheric pollutants because of its high oxidation ability (1). The toxic effects of photochemical oxidants have already been recognized by humans (2). Health effects associated with exposure to ambient ozone include reduced lung function, increased frequency of asthma attacks, school absences, increased emergency room visits and hospitalizations, and premature death (3). The task of reducing levels of exposure to ozone begins with an analysis to determine what levels of exposure are hazardous to human health and the environment, and then how can exposure to safe levels of ambient ozone be guaranteed. Once these problems are identified, ozone control strategies should be developed and implemented in order to prevent excessive risk to public health.

The abovementioned questions led to the concept of ozone guidelines and standards. The primary aim of the ozone guidelines is to provide a basis for protecting public health from the adverse effects of ozone and for eliminating ozone or reducing it to a minimum (4, 5). The ozone standard is the allowable ozone level in each area as defined by laws and regulations and is based on the ozone guidelines (6). As with other air pollutants, such as PM_2.5_, ozone guidelines or standards consist of three factors: level, average time, and form. Ozone guidelines and standards have been developed and gradually updated around the world. Developed countries and some international organizations have carried out systematic and effective research on controlling air pollution and formulating air quality guidelines and standards, accumulating rich experience in the process. The World Health Organization (WHO) and the United States (US) have a relatively complete process for the formulation and revision of guidelines or standards (7, 8).

This paper aims to review the process for the formulation and updates of ozone guidelines or standards by the WHO and the US, in order to excavate and analyze the scientific basis and reasons for ozone guidelines and standards and summarize the general rules and empirical methods for the formulation of ozone guidelines and standards. On the one hand, this would help other countries/localities in the world to better evaluate and revise guidelines and standards in the future. On the other hand, clarifying the rationale and concerns would guide the direction of scientific research related to guidelines and standards.

## 2. Air quality guidelines by the WHO

The main purpose of the WHO air quality guidelines (AQGs) is to provide a basis for protecting public health from the adverse effects of air pollution and for eliminating or reducing those pollutants to a minimum (9, 10). Ideally, guideline values should represent concentrations of chemical compounds in the air that would not pose any hazard to the human population. However, the realistic assessment of human health hazards necessitates a distinction between absolute safety and acceptable risk. Relevant information on ozone was carefully considered during the process of establishing guideline values, including the lowest observed adverse effect level (LOAEL) and safety or uncertainty factors (11). The AQGs for ozone were first proposed in 1987 and updated in 2000, 2005, and 2021 (Table 1). The changes in guideline values are based on an expert assessment of the available scientific evidence.

**Table 1:** Update of AQG of ozone by the WHO.

Update of AQG	Average time	Level (μg/m^3^)	Interim target (μg/m^3^)		Form
			1	2	
1987	1 h	150–200	—	—	The maximum of day value
	8 h	100–120	—	—	
2000	8 h	120	—	—	
2005	8 h	100	160		
2021	8 h^a^	100	160	120	A high percentile
	Peak season^b^	60	100	70	

a 99th percentile: the expected number of days per calendar year with maximum hourly average concentrations above the guideline is equal to 3 to 4 days.

b Average of daily maximum 8 h mean O_3_ concentration in the six consecutive months with the highest 6 months running-average O_3_ concentration.

### 2.1. The first edition of the European air quality guidelines

In 1958, the WHO recognized that air pollution was a threat to human health and well-being around the world. Before there was a risk to human health, the WHO began summarizing the situation and recommending preventive and remedial measures, culminating in the publication of the European AQGs in 1987. This first edition of the European *air quality guidelines* emphasizes the interpretation of the guidelines in terms of concentration and average time (12). Guidelines are based on the amount of ozone exposure that does not produce adverse health effects. The guidelines indicated a 1 h recommended O_3_ concentration of 150–200 μg/m^3^ (0.076–0.1 ppm). To reduce potential acute and chronic adverse effects and provide additional protection, guidelines give 8 h concentrations of 100–120 μg/m^3^ (0.05–0.06 ppm). The guideline values are based on numerous scientific findings on the health effects of exposure to concentrations higher than 200 μg/m^3^ O_3_. Changes in pulmonary function were associated with ozone exposure (235 μg/m^3^, 1–3 h) in healthy subjects during exercise (13, 14). Eye, nose, and throat irritation, chest discomfort, cough, and headache have been associated with hourly average oxidant levels above 200 μg/m^3^ (15).

### 2.2. Air quality guidelines—European update 2000

Advances in epidemiological research over the 1990s showed that adverse health effects may occur at much lower levels of ozone than identified previously. Epidemiological surveys found that respiratory symptoms, lung function decrease, worsened airway responsiveness, and airway inflammation were obvious at a concentration of 160 μg/m^3^ for an ozone exposure of 6.6 h in healthy adults (16). Field studies in children, adolescents, and young adults indicated that pulmonary function decrements can occur as a result of short-term exposure to ozone concentrations of 120–240 μg/m^3^ and higher (17). In 2000, the WHO updated the European *AQGs* to set the guideline value for ozone at an 8 h mean concentration of 120 μg/m^3^ (18). Health risk assessment demonstrated that acute effects on public health would be small with 8 h of ozone exposure at 120 μg/m^3^.

Another important point that needs highlighting is that the guideline limit time was adjusted to 8 h. Field studies have shown that health hazards associated with reduced O_3_ concentrations due to 1 h or 8 h exposure are very similar. Furthermore, a 1 h exposure to higher concentrations was comparable to an 8 h exposure at average levels to which people are usually exposed (19). Furthermore, the adverse health effects (i.e., lung function decrease and airway inflammation) would be better addressed by guidelines that limit average daily exposures, rather than a rare short-term air quality deterioration associated with abnormal weather. The 8 h guideline would protect against acute 1 h exposure in the current guideline and thus a 1 h guideline would not be necessary (18).

### 2.3. Air quality guidelines—global update 2005

Since the mid-1990s there has been no major addition to the evidence from chamber studies or field studies. However, epidemiological time series studies in low- and middle-income countries in Asia, where air pollution levels are highest, show significantly increased health impacts with ozone exposure (20). The WHO’s comparative risk assessment of ozone exposure quantified not only classical health effects, such as pathological changes in the cardiopulmonary system but also new health endpoints such as disease burden (increased morbidity and mortality) (21, 22). The combined evidence from these studies suggests a compelling, albeit small, positive relationship between daily mortality and ozone levels, independent of particulate matter (23). These studies have shown effects at ozone concentrations below 120 μg/m^3^, without clear evidence of a threshold (23, 24). Evidence from both laboratory and field studies also indicates considerable individual variation in exposure to ozone.

Accordingly, it is necessary to reduce the guideline from the existing level of 120 μg/m^3^. It is recommended that the AQG for ozone be set at 100 μg/m^3^ for a daily maximum 8 h mean ozone concentration according to the *AQGs*—*global update 2005* (25). This concentration will provide adequate protection to public health, though some health effects may occur below this level. There is some evidence that long-term exposure to ozone may have chronic effects but it is not sufficient to recommend an annual guideline. Besides, *AQGs*—*global update 2005* added O_3_ interim targets. These recommendations are incremental steps to gradually reduce air pollution and are intended to be used in heavily polluted areas. These targets are designed to facilitate a shift from high concentrations of air pollutants with serious health consequences to lower concentrations. The 8 h interim target value for ozone-1 (IT-1) was set at 160 μg/m^3^, at which transient lung function and inflammatory changes in the lungs were detected in young people undergoing intermittent exercise in the laboratory chamber (25).

### 2.4. Air quality guidelines—global update 2021

Since the publication of the *global update 2005*, many new studies have continued to document the adverse health effects of high levels of ozone in low- and middle-income countries, and much lower levels in high-income countries with relatively clean air (26–30). Furthermore, there was a great advance in expanding the global database of air pollution and exposure measurement as well as statistical analysis techniques and conceptualization of causal modeling in epidemiology. These studies reshaped the established exposure-response function of ozone and critical health outcomes (31, 32). Combined with the systematic review of the evidence and meta-analysis of quantitative effect, as well as assessment of the level of certainty of the evidence, the WHO revised the AQG level.

In the *global update 2021*, recommendations on the AQG of ozone consisted of long-term and short-term guidelines (8). A peak season ozone level of 60 μg/m^3^ (the average of daily maximum 8 h mean ozone concentrations) was recommended as a long-term guideline (8). The peak season is defined as the six consecutive months of the year with the highest 6 months running-average ozone concentration. The average daily maximum 8 h mean ozone concentrations of 100 μg/m^3^ were recommended as a short-term guideline. The short-term guideline was defined as the 99th percentile of the distribution of daily values (equivalent to three exceedance days per year) (33). The form of high percentiles provides some insulation from the impacts of special conditions that are conducive to maximum O_3_ formation. The reason for the high percentile rather than the maximum is that the maximum daily value for a given year is more volatile than the high percentile. The WHO has also updated the 2005 air quality interim targets to guide the implementation of the new AQGs.

For the development of long-term AQG levels, the systematic review on ozone and all non-accidental mortality reported a meta-effect estimate of relative risk =1.01 per 10 μg/m^3^ increase in the peak-season average of daily maximum 8 h mean ozone concentration (34). The average of the three lowest 5th percentile levels measured in these studies was the starting point for deriving an AQG level of 60 μg/m^3^ (35). The data obtained support a long-term, peak-season AQG level of no more than 60 μg/m^3^. For the development of short-term AQG levels, the systematic review on ozone for 8 h maximum concentration and all non-accidental mortality reported a meta-effect estimate of relative risk =1.0043 per 10 μg/m^3^ increase (36). The short-term AQG level is calculated using the ratio of 2 between the 99th percentile to the annual mean of 60 μg/m^3^, which gives a result of 120 μg/m^3^. Dividing this number by 1.24 gives a result of 97 μg/m^3^ (37), rounding to give a recommended short-term AQG level of 100 μg/m^3^. The data obtained support short-term, peak season AQG levels not exceeding 100 μg/m^3^.

By analyzing the WHO’s updating process of the ozone AQG, we observed a gradual improvement during the update process of the ozone guidelines. The perception of ozone health hazards changed from pathological changes in the cardiopulmonary system to increased risk of morbidity and mortality of cardiopulmonary disease, and other non-accidental diseases. In addition, the requirements for meeting guidelines have shifted from a single level to a more stable form of high percentile. In short, during the update of ozone guidelines, the improvement of ambient ozone monitoring and exposure assessment capabilities and the deepening awareness of the exposure-response relationship between ozone and adverse health effects could be possible main reasons for improvements in the guidelines.

## 3. US National Ambient Air Quality Standards

The Clean Air Act (CAA) directs the Environmental Protection Agency (EPA) to set National Ambient Air Quality Standards (NAAQS) for O_3_. NAAQS consist of a health-based “primary standard” judged to be requisite to protect public health with an adequate margin of safety and “secondary standards” to be requisite to protect public welfare including effects on crops, vegetation, buildings, visibility, and other (38). The CAA requires EPA to review the standards once every 5 years to determine whether revisions to the standards are appropriate (39). Reviewing NAAQS includes the following major steps: planning, comprehensive review, integrated scientific assessment, risk and exposure assessments, and employee policy assessments (40). The scientific review undertaken in the development of the Criteria Document and Staff Paper was thorough and extensive. Based on the scientific assessments, and the recommendations of the Clean Air Scientific Advisory Committee (CASAC) and public comments, the EPA administrator must judge whether it is appropriate to revise the standards (41). The EPA issued the first NAAQS for ozone in 1971 and revised the standards in 1979, 1997, 2008, and 2015 to ensure that NAQQS continues to protect public health and welfare (Table 2). The indicator, average time, level and form of O_3_ primary/secondary standards together would be appropriate to protect public health with an adequate margin of safety (7).

**Table 2:** Update of NAAQS of ozone by the US EPA.

Update of NAAQS	Average time	Level	Form
		Primary/secondary	
1978	1 h	0.12 ppm	1-expected-exceedance form^a^
1997	8 h	0.08 ppm	A concentration-based statistic form^b^
2008	8 h	0.075 ppm	
2015	8 h	0.07 ppm	

a The expected number of days per calendar year with maximum hourly average concentrations above 0.12 ppm is equal to or less than one.

b The 3 years average of the annual fourth-highest daily maximum 8 h average O_3_ concentration is less than or equal to the current level.

### 3.1. The first edition of NAAQS for O_3_

The EPA initially established primary and secondary NAAQS for photochemical oxidants in 1971. Both primary and secondary standards were set at an hourly average of 0.08 parts per million (ppm) total photochemical oxidants, not to be exceeded for more than 1 h per year. In 1979, the EPA completed its first periodic review of the criteria and standards for O_3_ and other photochemical oxidants (42). At the same time, EPA made significant revisions to the previous standard. The level of the primary and secondary NAAQS was changed to 0.12 ppm. The indicator was changed to O_3_. The form of standards was changed with the expected number of days per calendar year with a maximum hourly average concentration above 0.12 ppm to be equal to or less than one.

### 3.2. NAAQS update 1997

The EPA administrator revised the existing 1 h, 0.12 ppm primary standard with a new 8 h, 0.08 ppm primary standard on September 16, 1997, after carefully considering the information presented in the Air Quality Criteria Document for O_3_ and Other Photochemical Oxidants Update 1996 (43) and the Staff Paper, the advice and recommendations of CASAC, and public comments received on the proposal. The 8 h, 0.08 ppm primary standard will be met at an ambient air quality monitoring site when the 3 years average of the annual fourth-highest daily maximum 8 h average O_3_ concentration is less than or equal to 0.08 ppm (44). The guideline was appropriate to provide adequate and more uniform protection of public health from both short-term (1 to 3 h) and prolonged (6 to 8 h) exposures to ambient ozone. The current 1 h secondary standard was replaced by an 8 h standard that was identical to the new primary standard.

The 8 h average time was selected for the following reasons (45): (1) In the current NAAQS, the 1 h average time was initially selected primarily based on health outcomes associated with short-term (i.e., 1 to 3 h) exposure. There was only preliminary information on potential associations between long-term exposure and health outcomes considered qualitatively. (2) The new available information indicates that long-term (i.e., 6 to 8 h) exposure levels causing various health outcomes are lower than the current 1 h NAAQS level, and lower exposures are more frequent than higher exposures. (3) At lower O_3_ levels, adverse health effects were better associated with 8 h of exposure than an average time of 1 h (16, 46). (4) The quantitative risk assessment showed that the risks of short -and long-term exposure can be realized by a primary criterion with an average duration of 1 or 8 h. Therefore, there was no need to establish both 1 and 8 h standards. (5) While there was clear evidence of an association between long-term exposure and lung tissue damage in animal models, there was no corresponding evidence for human health. Therefore, it is not sufficient to select a longer time period as a criterion. Accordingly, an important public health policy consideration that an average time of 8 h results in a significantly more uniformly protective national standard than a 1 h standard supports the selection of an 8 h average time (47).

Since ozone does not have any discernible threshold, traditional paradigms of standard-setting cannot be applied in the usual way and risk assessment must play a central role in determining appropriate levels (31). The EPA administrator aimed to formulate a standard level that would sufficiently reduce risks since the CAA neither can nor requires a zero-risk standard. The existing 1 h standard set at 0.12 ppm O_3_ provided little margin of safety (48). At O_3_ exposure below 0.08 ppm for 8 h, the most established O_3_-related effects, although considered adverse, are transient and reversible. An 8 h standard set at 0.07 ppm would be closer to the peak background levels caused by precursor substances of non-human origin and are therefore not appropriate for some regions containing these sources. After carefully reassessing quantifiable public health risks, the EPA Administrator focused on the comparison between a recommended level of 0.08 ppm and an alternative standard setting of 0.09 ppm. According to the latest EPA risk assessment, setting the 0.09 ppm standard caused approximately 40% to 65% more health effects (moderate or substantial reductions in lung function and pain in children who play outdoors) than the 0.08 ppm standard in nine urban areas (49). Exposure assessment showed that the number of children exposed to 0.09 ppm for an average of 8 h was more than three times those exposed to 0.08 ppm in nine urban areas. While recognizing the uncertainties inherent in these estimates, the EPA Administrator claimed that the public health implications described in the proposal are significant enough to warrant a 0.08 ppm standard.

When evaluating alternative forms for primary standards, the adequacy of the public health protection provided is the most important consideration. Since 1979, the current form has raised concerns due to a lack of year-to-year stability. The impact of public health exposure to ambient O_3_ is related to the actual O_3_ level, not just whether the concentration is higher than the specified level. The CASAC recommended that concentration-based forms, within the range considered up to the fifth-highest concentration form, are appropriate for a health-based primary O_3_ standard. Concentration-based forms can minimize this instability and is protected from extreme meteorological events that contribute to O_3_ formation (50). Furthermore, due to the current difference between the safety boundaries of potential health effects provided by the range of the second to fifth-highest concentration forms not being sufficiently understood, it cannot be used as a basis for selecting the most restrictive forms (i.e., the second or third highest concentration forms). The proportion of monitoring sites reaching the fifth highest concentration standard and the number of days with the fifth highest concentration above the standard level both oppose the choice of this form. Therefore, the fourth highest concentration form will be combined with the 8 h average time and 0.08 ppm level to appropriately balance these public health factors (50). Given all this, the EPA Administrator determined to establish a revised 8 h, 0.08 ppm standard with a form of the 3 years average of the annual fourth-highest daily maximum 8 h average O_3_ concentrations.

### 3.3. NAAQS update 2008

After an extensive review of several scientific studies on the impact of ambient ozone on public health and the environment, the US EPA significantly strengthened NAAQS for ozone and revised the 8 h primary and secondary standards to a level of 0.075 ppm (51, 52). In addition to changing the standard level, the EPA also assigned the standard level to three decimal places. The revised standards would be met when the 3 years average of the annual fourth-highest daily maximum 8 h average is less than or equal to 0.075 ppm. These revisions were proposed to provide increased protection for children and other “at risk” populations against an array of O_3_-related adverse health effects. These effects range from decreased lung function and increased respiratory symptoms to emergency department visits and hospital admissions, and possibly cardiovascular-related morbidity, as well as total non-accidental mortality (53).

We noticed that the main revision was the strengthened level of O_3_ in the 2008 update. There was a strong body of clinical evidence of lung function decrements, respiratory symptoms, and other airway responses at O_3_ exposure above levels of 0.080 ppm (54). Epidemiological evidence indicated the observation of a wide range of serious health effects, including emergency department visits, hospital admissions, and premature mortality, with an ozone concentration below 0.080 ppm (19). Based on the evidence and CASAC’s recommendations, the EPA Administrator recommended that a standard in the 0.070 to 0.075 ppm range be necessary to protect public health. The likelihood of obtaining benefits to public health with a standard set below 0.075 ppm O_3_ decreases, so it was not suggested that a lower standard was needed to provide this degree of protection. Accordingly, a standard set at 0.075 ppm would be sufficient to protect public health with an adequate margin of safety.

We noticed that cost-benefit analysis had an important role in choosing the level of the standard. To estimate the benefits of meeting a standard, the EPA uses a sophisticated peer-reviewed approach to model the relationship between air quality and health and welfare effects and the dollar values of resulting public health improvements. The EPA estimated that the revised standards would yield health benefits valued between US$2 billion and US$17 billion (55). Those benefits include preventing cases of bronchitis, aggravated asthma, hospital and emergency room visits, non-fatal heart attacks, and premature death. To estimate the cost of meeting a standard, the EPA uses several peer-reviewed methods to model the cost of control measures needed to reduce ozone precursor. The EPA estimated that the cost of implementing a 0.075 ppm standard would range from US$7.6 billion to US$8.8 billion per year by 2020 (55).

### 3.4. NAAQS update 2015

In 2015, the US EPA further strengthened the NAAQS value for ambient ozone to 0.07 ppm based on extensive scientific evidence (56). The updated standards would improve public health protection, especially for at-risk groups, including children, older adult, people of all ages with lung diseases such as asthma, and people who are active outdoors, especially outdoor workers (57). This would also improve the health of trees, plants, and ecosystems. When determining the updated standards, the EPA Administrator considered factors such as the nature and severity of health effects, the size of the at-risk groups affected, and the degree of uncertainty on ozone-related health effects (58). The law obliges the EPA Administrator to set “necessary” standards—neither more nor less than necessary—to achieve this goal.

Based on an expanded body of scientific evidence, the 75 parts per billion (ppb) standard in 2008 was not requisite to protect public health with an adequate margin of safety, as required by law. These studies clearly showed that 72 ppb of ozone may be harmful to healthy exercising adults (59). A standard of 70 ppb is below the level shown to cause adverse health effects in clinical studies. The evidence that health effects were harmful or “adverse” in several clinical studies in which some adults were exposed to ozone at levels as low as 60 ppb was uncertain (19, 60). More importantly, the EPA Administrator focused on children’s exposure, particularly repeated exposures (61). Repeated exposures are important, because the more times children are exposed to ozone, the more likely they will experience serious health effects. Children are at increased risk from ozone exposure and are more likely to be active outdoors when ozone levels are high. A standard of 70 ppb would protect 99.5% of children from even single exposures to ozone at 70 ppb as well as more than 98%of school-age children from repeated exposures to ozone concentrations as low as 60 ppb, which would mean a 60% improvement over the current standard (58). The updated standard of 70 ppb would protect at-risk populations with an adequate margin of safety. Furthermore, the public health benefits of the updated standard would reach US$2.9 to US$5.9 billion by 2025, exceeding the estimated $1.4 billion cost (62).

By reviewing the process undertaken by the US EPA to revise ozone NAAQS, we realize that the US has a rich experience in standard development. For each NAAQS update, the EPA scientifically reviewed and demonstrated the level, form, and average time of O_3_ standards, and was open to public discussion and opinion. The form and average time of the standards were established earlier and have been used ever since by the EPA. We found that the progressively tightened ozone standard levels relied not only on the perceived health hazards of ozone but also on the assessment of health and welfare benefits under current standards. These are based on surveys of relationships between ozone exposure and human health endpoints as well as accurate benefit-cost models of ozone compliance, exposure and risk assessment, and pubic opinion.

## 4. Summary and conclusion

In essence, ozone guidelines and standards are completely different concepts. Guidelines are mainly the limit at which ozone does not pose a health hazard (an acceptable range of health hazards) (63), whereas standards are set to limit the level of ambient ozone to protect human health and public welfare (64). After reviewing the WHO’s AQG and the US EPA’s NAAQS, we found that the revision of guidelines is mainly based on exposure-response relationships between O_3_ exposure and adverse health effects. The NAAQS need to be more comprehensive, not only based on the health hazards but also on a comprehensive consideration of exposure assessment, risk assessment, compliance cost and benefit analysis, and public opinion. We suggest that these could be the possible empirical methods for the formation of ozone guidelines and standards. Of course, the WHO’s AQG and the US EPA’s NAAQS have something in common. In both AQG and NAAQS the indicators time, level, and form are used as the four elements of the limit value. Ozone, the main photochemical oxidant, is used as the indicator, and both use 8 h as a time limit. AQG adopt the form of high percentile (see section 2.4); NAAQS take a concentration-based statistic form (see section 3.2). For the comparable O_3_ limits level of 8 h, the current AQG limit is 100 ug/m^3^ (equivalent to 50 ppb), while the EPA currently limits NAAQS to 70 ppb. This difference in level reflects the difference in the basis for the revision of the guidelines and the standards.

Regardless of revisions, the recognition of the adverse health effects caused by ozone is one of the important bases for guidelines and standards. In the previous section, the description of the adverse health effects of ozone was fragmented and incomplete. Here, we summarize the possible adverse effects on human health caused by ozone exposure that is close to threshold/limits, especially focusing on the key evidence used now or in the future for revision of guidelines and standards (Table 3). More studies have illustrated the hazards of ozone in terms of increased risk of health endpoints (disease burden, such as morbidity and mortality) from increased ozone exposure, without distinguishing ozone exposure levels (31, 32). These studies pointed out that there was no threshold for ozone health hazards. Even if a threshold exists, it should be near the background ozone level. Recent studies have found a significant association between ozone exposure as low as 40–50 ppb and daily mortality (65). Although the evidence remains to be verified, there is a possibility of tightening current ozone standards.

**Table 3:** The possible adverse effects on human health caused by ozone exposure that is close to the threshold/limits.

Health endpoints	Description	Level	Location
Pulmonary function (16)	Significant decrease	0.08 ppm	North Carolina, United States
Pulmonary function (59)	Significant decrease in FEV1	70 ppb	Carolina, United States
Pulmonary inflammation (60)	Significant increases in polymorphonuclear leukocyte	60 ppb	Carolina, United States
Daily total mortality (31)	Increased risks	Higher than 100 μg/m^3^	Jiangsu, China
Daily mortality (65)	Significant associations	Higher than 40, 50 ppb	Eastern Asian Countries

In particular, we noticed that the studies analyzing the health effects of ozone on which the guidelines and standards are based only considered the effects of ozone. At present, the world frequently registers air composite pollution of PM_2.5_ and O_3_ (66). Mixed chemicals in the air, can have additive, synergistic, or antagonistic health effects, however, knowledge of these interactions is still rudimentary. More and more studies have begun to consider the effects of combined exposure to PM_2.5_ and O_3_ (67). Recent studies showed that there were remarkably synergistic interactions between PM_2.5_ and O_3_ on non-accidental, cardiovascular, and respiratory mortality, suggesting that combined exposure to PM_2.5_ and O_3_ could further exacerbate their single-pollutant health risks (68). These studies would inevitably serve as an important reference basis for revising the ozone guidelines and standards. The WHO also acknowledged the need to develop comprehensive models to quantify the effects of multiple exposures on human health (8). So more joint exposure studies are needed to reshape the exposure-response relationship between ozone and adverse health effects, and further support the development of ozone guidelines and standards.

This paper reviews the revision process adopted by the WHO on AQG and the US EPA on NAAQS and analyzes their revision basis and empirical methods. We found that a large number of epidemiological and toxicological studies and exposure-response relationships provided strong support for the revision of the ozone guidelines. When setting the standard, the ozone pollution situation for a particular region was effectively considered, and the costs and benefits of meeting the revised ozone standard were reasonably estimated. Accordingly, the epidemiological and toxicological studies and establishment of exposure-response relationships, as well as benefit-cost models and exposure and risk assessments should be strengthened for the further update of guidelines and standards. Furthermore, with the increasing prominence of combined air pollution, more combined (especially ozone and PM_2.5_) exposure research related to ozone guidelines and standards should be conducted.

## Author contributions

KX: Conceptualization, Writing – original draft. XZ: Supervision, Writing – review & editing.
